# Severe paravalvular detachment of a bioprosthetic aortic valve in atypical infective endocarditis

**DOI:** 10.1093/ehjcr/ytag182

**Published:** 2026-03-12

**Authors:** David Füller, Oliver Ritter, Vadim Tchaikovski

**Affiliations:** Department of Internal Medicine, Division of Cardiology, Nephrology, and Pulmonology, University Hospital Brandenburg an der Havel, Brandenburg Medical School (Theodor Fontane), Hochstraße 29, D-14770 Brandenburg an der Havel, Germany; Department of Internal Medicine, Division of Cardiology, Nephrology, and Pulmonology, University Hospital Brandenburg an der Havel, Brandenburg Medical School (Theodor Fontane), Hochstraße 29, D-14770 Brandenburg an der Havel, Germany; Department of Internal Medicine, Division of Cardiology, Nephrology, and Pulmonology, University Hospital Brandenburg an der Havel, Brandenburg Medical School (Theodor Fontane), Hochstraße 29, D-14770 Brandenburg an der Havel, Germany

## Case description

A 68-year-old man, 16 years after biological aortic valve replacement, presented in the Emergency Department with progressive exertional dyspnoea, currently on minimal exertion (NYHA III), and progressive lower leg oedema since 1 week. Prior to recent events, the patient has been in good functional status. He has just returned from a trip to Hawaii. N-terminal proBNP was significantly elevated (3926 pg/mL, reference value <125 pg/mL). C-reactive protein was moderately elevated (36.5 mg/L, reference value <5 mg/L). Transthoracic echocardiography revealed at least moderate aortic valve prosthesis insufficiency with a large eccentric insufficiency jet.

**Figure 1:**
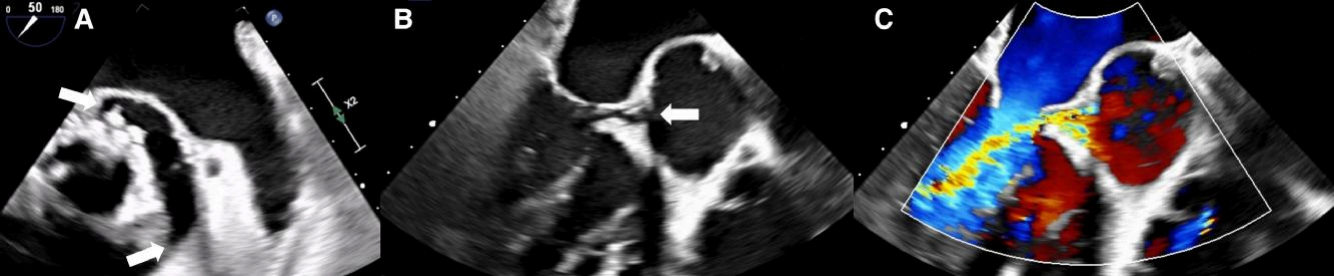
(*A*) Severe paravalvular detachment of about 120° of the circumference of the bioprosthetic aortic valve, short axis. (*B*) Valve dehiscence, three-chamber view, long axis. (*C*) Severe paravalvular insufficiency, colour Doppler, three-chamber view, long axis.

Transthoracic and transoesophageal echocardiography confirmed an extremely hypermobile prosthesis with detachment of about 120° of its circumference (*Figure 1A* and *B*) and hypermobility of about 50–60° (see Supplementary material online, *Video S1*), leading to severe paravalvular insufficiency (*Figure 1C*; Supplementary material online, *Video S2*). The patient was transferred for urgent replacement of the valve prosthesis.

Intraoperative findings confirmed endocarditis. Multiple blood cultures have remained negative. Polymerase chain reaction on intraoperative tissue samples identified *Cutibacterium acnes*, a relatively rare cause of endocarditis in general (approximately 3%), predominantly affecting men and prosthetic valve recipients.^1^ Its clinical presentation often involves mild or even absent inflammatory signs and negative blood cultures.^2^  *C. acnes* is an anaerobic Gram-positive bacterium. It is the most abundant bacterium of the human skin microbiome since adolescence, participating in skin homeostasis, but it can act as an opportunistic pathogen. An important virulence factor is the formation of a biofilm. Late-onset bioprosthetic valve endocarditis from *C. acnes* 5 years after composite aortic root and valve replacement has previously been reported by others,^3^ but no case of as long as 16 years between bioprosthetic valve implantation and endocarditis has been reported in the literature. This case also highlights a rare differential diagnosis for pulmonary embolism for a patient presenting with acute-onset dyspnoea following a long-haul flight.

## Supplementary Material

ytag182_Supplementary_Data

## Data Availability

No datasets were generated or analysed beyond those included in this published article. Further information is not publicly available due to patient privacy.

## Notes

**Consent:** The explicit written consent of the patient to report on his case has been obtained by the authors.
