# Supplementary material for: Severe paravalvular detachment of a bioprosthetic aortic valve in atypical infective endocarditis
Source: Eur Heart J Case Rep. 2026 Mar 12;10(3):ytag182. doi: 10.1093/ehjcr/ytag182 (PMC13012214; doi:10.1093/ehjcr/ytag182)
Supplement: ytag182_Supplementary_Data [file ytag182_supplementary_data.zip › Supplementary_Legends.docx]

Supplementary video 1: Hypermobility of the bioprosthetic aortic valve, three chamber view, long axis.

Supplementary video 2: Hypermobility of the bioprosthetic aortic valve, color doppler, three chamber view, long axis.
